# Case Report: Remission of a patient with complex combination of autoimmune diseases by anti-CD19 CAR-T cell therapy

**DOI:** 10.3389/fimmu.2025.1645304

**Published:** 2025-08-21

**Authors:** Xudong Liu, Wenxiang Zhu, Nan Su, Yanyi Ma, Fang Wang, Jingyi Xu, Heyang Zhang, Xiaomeng Luo, Yuetong Zhao, Jianxun Wang, Yuanyuan Shi, Yishuo Li, Pingting Yang

**Affiliations:** ^1^ Department of Rheumatology and Immunology, The First Hospital of China Medical University, China Medical University, Shenyang, Liaoning, China; ^2^ Nanozyme Laboratory in Zhongyuan, Henan Academy of Innovations in Medical Science, Zhengzhou, Henan, China; ^3^ Department of Hematology, The First Hospital of China Medical University, China Medical University, Shenyang, Liaoning, China; ^4^ Shenzhen Cell Valley Biomedical Co., LTD, Shenzhen, Guangdong, China

**Keywords:** autoimmunity, systemic lupus erythematosus, antiphospholipid syndrome, type B insulin resistance syndrome, anti-CD19 CAR-T

## Abstract

CAR−T cell therapy has been proven effective in various autoimmune diseases, with most studies utilizing lentiviral‐transduced CAR−T cells. In recent years, retroviral vector−transduced CAR−T cells—characterized by a high positivity rate, stable cell lines, and lower plasmid requirements—have attracted increasing attention. This article presents a complex case of a patient with SLE combined with APS and TBIRS. For four years following the diagnosis, the patient underwent conventional steroid therapy and immunotherapy, which yielded unsatisfactory and relapse−prone results. After receiving anti−CD19 CAR−T cells transduced with a retroviral vector, the patient experienced an excellent postoperative recovery without any infusion−related adverse reactions. Post−treatment, the patient’s creatinine, anti-dsDNA antibodies, albumin, and glycated hemoglobin levels returned to normal, eliminating the need for ongoing glucocorticoids or hypoglycemic agents. Although there are some available reports of CAR-T cells treating SLE, it is still very rare and significant for successfully treating such a complicated case, especially after proving the unavailability of traditional therapy. Furthermore, this is the first reported case of treating TBIRS syndrome with retroviral vector−transduced CAR−T therapy.

## Introduction

Systemic lupus erythematosus (SLE) is a diffuse, systemic autoimmune disease characterized by the production of autoantibodies, which can affect multiple organs and systems, including the kidneys, heart, lungs, skin, and others (1, 2), causing inflammation and organ damage (3). Currently, dysregulated B-cell activation is considered a key factor in the development of SLE. It can be triggered by multiple factors, including genetic and environmental factors (4). SLE is more common in women and non-White populations (3). The long-term mortality rate of SLE remains high, with a 20-year survival rate after diagnosis of approximately 78% (1). Causes of death include organ damage, damage caused by glucocorticoid therapy, and cardiovascular disease (1). Among the complications of SLE, lupus nephritis (LN) is the most common, occurring in 25-60% of SLE patients, and it is also the leading cause of morbidity and mortality (5). Approximately 10-30% of LN patients progress to end-stage kidney disease (1, 5).

Antiphospholipid syndrome (APS) is a rare autoimmune thrombotic disorder characterized by the presence of antiphospholipid antibodies, such as anticardiolipin antibodies and anti-β2-glycoprotein I antibodies (6). APS can manifest in various clinical forms, including venous, arterial, and microvascular thrombosis, as well as obstetric complications (6). APS is closely associated with SLE, with approximately 40% of SLE patients also having APS. Similarly, a significant proportion of primary APS patients test positive for ANA or anti-dsDNA antibodies (7).

Type B insulin resistance syndrome (TBIRS) is a clinical condition caused by the presence of autoantibodies against the insulin receptor (8, 9). Its typical characteristics include extreme insulin resistance, diabetes that does not respond even to high doses of insulin, significant weight loss, severe hyperandrogenism, and acanthosis nigricans (8, 10, 11). TBIRS is a rare autoimmune disease, with just over 100 cases reported as of 2024 (9). TBIRS is strongly associated with other autoimmune disorders; the literature most notably links it with systemic lupus erythematosus (SLE). In a 28-year follow-up study, 11 out of 24 (46%) of TBIRS patients were also diagnosed with SLE. TBIRS is highly dangerous, with a reported 10-year mortality rate of up to 54% (12). This high mortality is largely attributed to the severity of the underlying systemic disease, although confirmed disease-related deaths have been associated with episodes of hypoglycemia.

The current standard treatment for SLE involves either high-dose glucocorticoid pulse therapy (up to 1 g/day intravenously for four consecutive days) or conventional-dose glucocorticoid therapy, typically combined with immunosuppressants (3, 9, 13). However, glucocorticoids—especially when used long-term at high doses—can cause significant side effects. These include Cushing’s syndrome, osteoporosis, and avascular necrosis, as well as an increased risk of infections (14) (with the risk further heightened when used in combination with immunosuppressants). Significant infections may occur even at a median oral prednisone dose of only 7.5 mg, and for every additional 10 mg of prednisone, the risk of infection reportedly increases 11-fold (15). Furthermore, due to individual differences, some patients do not respond adequately to these treatments and frequently experience relapses (1, 16). Additionally, under traditional treatment regimens, patients must remain on lifelong medications to maintain their condition.

Case reports on CAR-T cell therapy for the treatment of SLE began to emerge in 2021, including cases of relapsing disease (17) and acute lupus nephritis triggered by hemodialysis (18). In 2024, the first report of CAR-T therapy being used to treat pediatric SLE was published (19). Beyond individual case reports, case series on SLE, IIM, and SC (20), as well as a Phase I clinical trial summarizing the use of BCMA-CD19 CAR-T cell therapy for SLE (16), have also been published. However, these reports primarily focus on relatively homogeneous cases of SLE with comorbid lupus nephritis (LN), with only one case involving changes in partial examination indices after CAR-T therapy for SLE combined with APS (21). None of these reports addresses cell therapy for TBIRS.

Here, we present a complex case in which the patient was simultaneously diagnosed with SLE, LN, APS, and TBIRS, and also had a history of pulmonary tuberculosis. When conventional steroid pulse therapy, as well as immunotherapy, failed to achieve lasting control, resulting in recurrent conditions, the patient underwent anti−CD19 CAR−T cell therapy. Compared to previous treatments, CAR−T therapy achieved a remission of SLE and APS, and also a remarkable cure for TBIRS, eliminating the need for ongoing maintenance with glucocorticoids, anticoagulants, or hypoglycemic agents. This case is the first to include the syndrome of TBIRS in conjunction with APS, LN, and SLE.

## Method

We have encoded a novel anti-CD19 chimeric antigen receptor (CAR) using the MFG retroviral vector. In this construct, immediately following the LTR5 sequence, the CAR contains anti-CD19 single-chain variable fragments (scFv). These scFv are derived from FMC63, with its heavy and light chain variable regions connected by a (G4S)^3^ linker. It is followed by a CD8 hinge region and the corresponding transmembrane domain, then by an intracellular CD28 co-stimulatory domain, and finally by the CD3ζ T-cell activation domain ending with the LTR3 sequence (**Supplementary Figure S1A**). For retroviral packaging, we employed a two-step process. First, we used Phoenix-ECO cells to introduce the retroviral vector, and subsequently, we infected PG13 cells to establish a stably transduced cell line. We then harvested the retroviral vector from this stable cell line for the transduction of human T cells that have been activated in advance. All the viral vectors were packaged in the GMP-grade production facility at Shenzhen Cell Valley and were released after passing mycoplasma/chlamydia quality control.

For the preparation of CAR-T cells, the process begins by isolating the patient’s autologous peripheral blood mononuclear cells (PBMCs) from whole blood. These cells are then forwarded within four hours to a class C clean production facility at Shenzhen Cell Valley, where all subsequent processing is conducted. Next, T cells are enriched from the PBMCs using CD3+ magnetic beads and activated with CD3/CD28 antibodies for two days. After activation, the cells are transduced with previously harvested retroviral vectors to express chimeric antigen receptors. Flow cytometry is used to assess transduction efficiency, while qPCR quantifies the number of integrated retroviral copies per T cell. Finally, the CAR-T cells are expanded in an IL-2-supplemented environment until they reach the appropriate dose for patient infusion. The expansion process took about 21 days.

After passing quality control and meeting batch release standards, the cells were resuspended in DMSO-free injectable saline (0.9% NaCl) supplemented with human albumin before encapsulation. The final product was transported at 2–8°C to the First Hospital of China Medical University within two hours. Upon arrival, the cells were subsequently injected into the patient at a dose of 2 × 10^6^ cells per kg, including 1 × 10^6^ CAR-T cells per kg. (**Supplementary Figure S1B**).

## Case presentation

**Figure 1:**
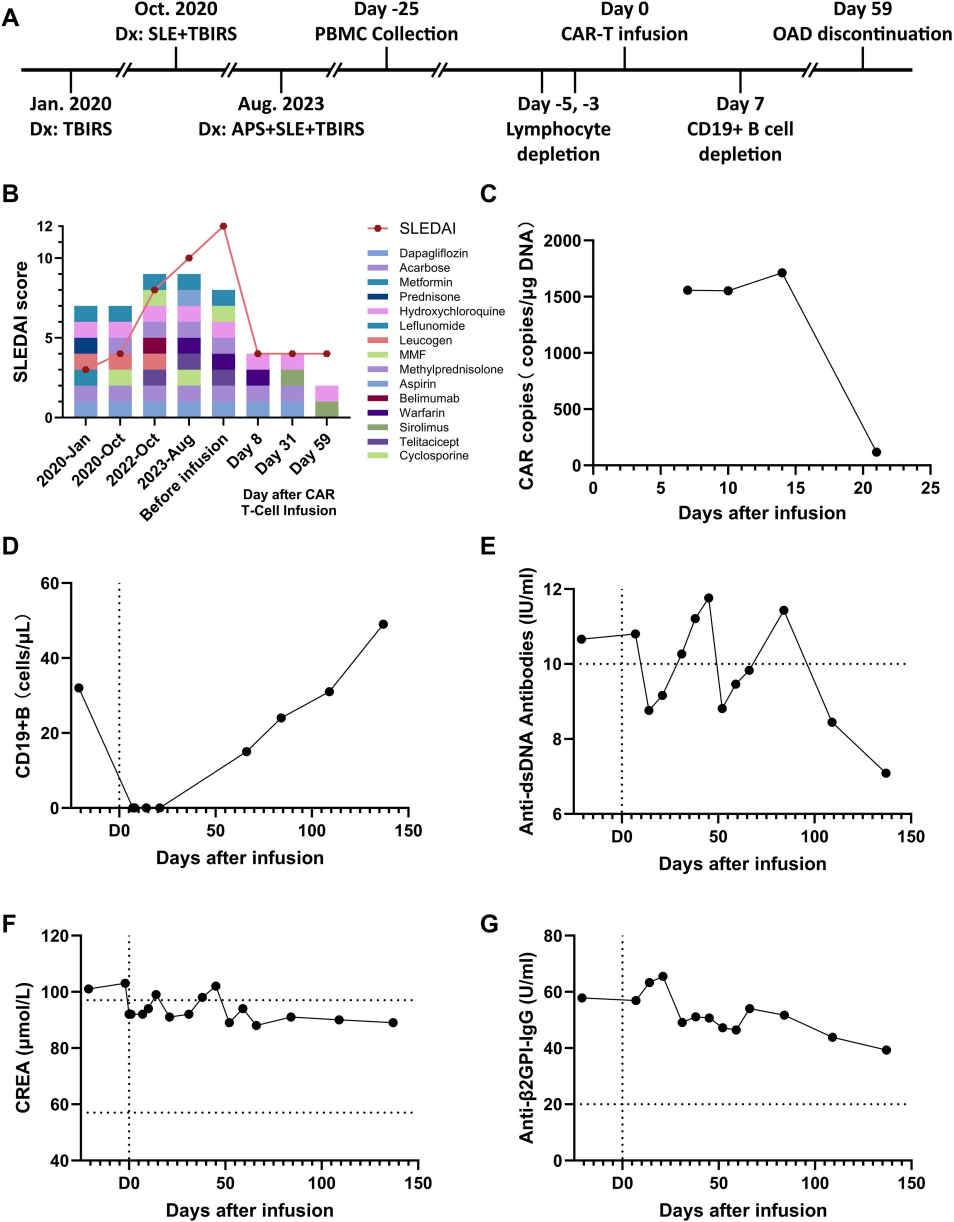
Evolution of immunological and metabolic parameters under treatment. **(A)** Timeline and protocol of diagnosis and treatment. **(B)** Changes in the patient’s medication and his SLEDAI score, **(C)** Number of CAR copies in the patient’s total DNA after cell infusion. **(D)** The patient’s CD19+ B cell counts in the peripheral blood. **(E)** Changes in the patient’s level of anti-double-stranded DNA antibodies. **(F)** Changes in the patient’s creatinine (CREA) level. **(G)** drop in the patient’s level of anti-β2-glycoprotein I antibodies.

In 2020, 4 years before infusion, the patient presented to the endocrinology department with a complaint of thirst, polydipsia, polyuria for six months, aggravated for one week, and sudden emaciation. Black acanthosis was seen in the perianal, axillary, and inguinal regions. The patient was emaciated, and OGTT and islet function showed that the patient was hyperinsulinemic and insulin-resistant (see **Table 1** for OGTT results). Further laboratory tests revealed ANA1 (+), ANUA (+), WBC (2.06 * 10^9/L), NE (1.24 * 10^9/L), C3 (0.34g/L), and C4 (0.05g/L), which were consistent with the diagnosis of SLE. The patient presented with refractory hyperglycemia, acanthosis nigricans, hyperandrogenism, and SLE, leading to a final diagnosis with a special type of diabetes categorized as TBIRS with SLE. The detailed timeline of the disease progression is summarized in **Figure 1A**.

**Table 1:** OGTT test of the patient in 2020, before treatment.

OGTT	0min	30	60	120	180
GLU(mmol/L)	10.60	12.67	17.24	19.95	21.83
CPS(pmol/L)	3267.60	4458.70	5024.30	5311.10	5530.70
IRI(mIU/L)	503	518.5	650	690	701

GLU, Blood glucose concentration; CPS, Serum c-peptide concentration; IRI, Serum insulin concentration.

The patient was then put on a strict diet for diabetes mellitus, lifestyle intervention, and was given dapagliflozin, acarbose, and metformin. As for systemic lupus erythematosus, he was given prednisone and hydroxychloroquine. After 4 months of treatment, the patient developed tuberculosis and stopped taking the medication for 2 months due to quadruple anti-tuberculosis treatment.

3 years ago for the follow-up examination, the laboratory results suggested that the urine protein index was (2+), therefore the treatment with mizoribine, hydroxychloroquine, cyclosporine (for 2 months), moratorium mescaline, and Belimumab (for 3 months) was initiated, with follow-up examinations performed every two months during meditation. No improvement was observed in the patient’s condition during the time, belimumab was discontinued, and switched to ixekizumab. 22 months ago, the patient was infected with COVID-19. Platelets were progressively reduced, and no significant improvement was observed after continuing the above-mentioned treatment.

One year ago, the patient was hospitalized due to the sudden onset of fundus hemorrhage. The platelet number dropped to a minimum of 1*10 (9)/L. Relevant laboratory tests showed that both aCL and β2GP1 antibodies were positive; thus, he was diagnosed with antiphospholipid syndrome (APS). The patient was given a temporary platelet transfusion and symptomatic treatment for hemostasis. Methylprednisolone 250 mg IV infusion QD x 4 days was performed, followed by oral dexamethasone, cyclosporine, and tetracycline. The patient was discharged from the hospital after the condition improved, but the platelets were still lower than normal. Upon discharge, warfarin was prescribed. Regular follow-up after discharge showed the platelet number remained at 26-69*109/L (mostly 40-50*109/L), urinary protein remained 2+ to 3+. According to the doctor’s instructions to adjust the medication, the current treatment is methylprednisolone, moratorium, and tetraciprazole. The treatment was unsatisfactory as reported by the patient.

Due to recurrent conditions and poor results of conventional medication and immunotherapy, the patient decided to undergo CAR-T cell transfusion therapy and underwent lymphocyte mono-colonization and related lab tests from 2024.09.23-2024.09.26. At this time, the patient exhibits dense, skin-colored papules ranging in size from a needle tip to a millet seed on both wrists, elbows, knees, ankles, and the extensor sides of the feet. The patient also experiences hair loss and frequent oral ulcers, the condition of which spontaneously improves after one week but recurs 2–3 days afterward.

The patient’s lymphocyte was collected on 2024-09-26. On October 16, 2024, the patient started lymphodepletion therapy using fludarabine phosphate and cyclophosphamide, with daily monitoring of CBC, CRP, coagulation, and liver/kidney function. On October 21, 2024, the infusion of 50 mL (total 1.56E+08 cells) anti-CD19 CAR-T cells was performed smoothly (**Supplementary Figure S1B**). After CAR−T infusion, the regimen was changed to: Levetiracetam, Isoniazid, Famciclovir, Co−trimoxazole, and Fluconazole. Treatment for TBIRS with SLE has continued with dapagliflozin, acarbose, hydroxychloroquine, and warfarin.

After CAR-T cell infusion, the patient’s white blood cell and neutrophil counts decreased. (**Supplementary Figure S1C**) Serum levels of CRP and IL-6 increased, and CRS did not occur (**Supplementary Figures S1D, E**). During the observation and follow-up periods after CAR-T infusion, The CAR copy in the patient’s total DNA remained at a high level at day 15 (**Figure 1C**), and the number of B cells has been cleared for over 3 weeks (**Figure 1D**), and the levels of dsDNA and CREA returned to normal (**Figures 1E, F**). The medication was further reduced to only hydroxychloroquine and sirolimus for the treatment of SLE (**Figure 1B**). In response to symptoms of APS, the patient’s Anti-β2GPI-IgG antibody showed a continuous downward trend (**Figure 1G**), and the PT-INR index returned to the normal range (**Supplementary Figure S1F**). In response to symptoms of TBIRS, the patient’s fasting blood glucose remained within the normal range, avoiding the occurrence of hypoglycemia (**Supplementary Figure S1G**). The glycated hemoglobin level gradually returned to normal over the following two months (**Supplementary Figure S1H**), with the use of dapagliflozin and acarbose gradually discontinued. For the symptoms of LN, the plasma albumin concentration also returned to normal and never exceed from the normal range during follow-up examinations. (**Supplementary Figure S1I**). After four months of regular follow-up visits, it is confirmed that the patient’s TBIRS was completely relieved with no signs of recurrence.

## Discussion

Currently, the conventional standard treatment includes rituximab, cyclophosphamide, and pulse therapy with glucocorticoids (3, 9, 22). Monoclonal antibody drugs like rituximab, or belimumab, used in this case, are used to reduce CD19 B lymphocyte levels (5, 23–25). Indeed, the dysregulation of B lymphocyte activation and proliferation is a significant factor in the pathogenesis of SLE and all of its related syndromes (4). However, these two monoclonal antibody drugs have minimal effects on plasma cells. As a result, SLE patients exhibit significant differences in tolerance to these monoclonal antibody drugs (24, 26), making it impossible to achieve sustained drug-free remission. Even though telitacicept possesses additional APRIL inhibition ability (27), it was still unable to help slow the progression of the disease in this case, and lifelong medication remains necessary.

In addition, following this regimen, patients must continue long-term treatment with high doses of glucocorticoids (>10 mg per day) and immunosuppressants. The side effects of glucocorticoids—especially with prolonged use—are well documented and include Cushing’s syndrome, osteoporosis, and avascular necrosis (14, 15). Continuous immunosuppressant therapy further increases the risk of infections, placing a significant burden on the body, making it one of the important reasons for mortality on a long-term scale of SLE patients, not to mention that 50% of patients will relapse within 12 months (1).

Furthermore, with the additional diagnosis of APS, the patient needs lifelong anticoagulation therapy (28). However, anticoagulant treatment increases the risk of bleeding—studies have reported that up to 40% of patients experience at least one bleeding episode within a year (29). Due to SLE, the patient had already developed progressive thrombocytopenia, and the addition of oral warfarin further elevated the risk of hemorrhagic complications (30). In this case, when the patient attempted to discontinue glucocorticoid intake, he developed life-threatening severe thrombocytopenia along with funduscopic hemorrhage. Although high-dose glucocorticoid pulse therapy was administered as a first aid, it only provided temporary symptom relief and never a complete remission. Following that treatment, the patient still required long-term glucocorticoids, anticoagulants, immunosuppressants, and hypoglycemic agents, with no hindrance to any of the diseases from progression.

In contrast, after receiving autologous CAR−T cell therapy, the patient experienced no postoperative adverse reactions and was able to recover within a week. After treatment, the patient’s creatinine and anti-double-stranded DNA antibody levels returned to the normal range, indicating effective SLE treatment (**Figures 1E, F**). Urine protein levels continued to decrease (from ++++ to +), urinary microalbumin levels dropped to around 400 mg/L, hematuria was negative, and urinary red and white blood cell counts returned to normal. Plasma albumin levels were restored to normal (**Supplementary Figure 1I**), indicating reduced kidney involvement and effective LN treatment. Anti-beta2GPI levels continued to decline (**Figure 1G**), no new thrombotic events occurred, and the patient’s coagulation parameters returned to normal, demonstrating the restoration of coagulation function. The HbA1c levels steadily decreased and eventually stabilized within the normal range (**Supplementary Figure 1H**). Consequently, the patient no longer needed to continue treatment with glucocorticoids, anticoagulants, or hypoglycemic medications, and the intake of immunosuppressants was reduced to a minimum and the possibility of recurrence has not been observed until now (**Figure 1B**).

Although CAR−T therapy for autoimmune diseases is not entirely new, previous studies and case reports have mostly focused on SLE with LN, only one case mentioned SLE with APS, and no patients in those reports had been diagnosed with TBIRS. This case is the first to specifically focus on TBIRS—an autoimmune diabetes syndrome—in conjunction with APS, LN, and SLE, highlighting its presentation and treatment outcomes.

In conclusion, this paper presents a rare case of SLE complicated by APS and TBIRS, where conventional therapies failed to prevent disease progression and the patient experienced relapses. After CAR-T cell therapy, the patient showed significant improvements, including reduced creatinine, normalized anti-dsDNA antibodies, decreased urinary protein, resolved thrombosis, and stable glycated hemoglobin levels without the need for lifelong corticosteroids or anticoagulants. This case highlights CAR-T therapy as an effective treatment for complex autoimmune conditions and demonstrates the advantages of retroviral vector-based CAR-T cells.

## Data Availability

The original contributions presented in the study are included in the article/**Supplementary Material**. Further inquiries can be directed to the corresponding authors.

## Supplementary material

Supplementary Figure 1
**(A)** CAR construct used in the case. **(B)** CAR-T cell infusion protocol for the patient. **(C)** Monitoring of concentrations of white blood cells (WBC), neutrophils (NE), and lymphocytes (LY) in blood over time. **(D)** The patient’s concentration of C-reactive protein (CRP) in blood. **(E)** Monitoring of the patient’s concentration of interleukin-6 (IL-6) in blood. **(F)** Monitoring of the patient’s prothrombin time-international normalized ratio (PT-INR) before and after reinfusion over time. **(G)** The patient’s fasting plasma glucose (FPG) concentration over time. **(H)** Patient’s HbA1c concentration over time. **(I)** Monitoring of the patient’s serum albumin level.
